# Glucagon enhances subcutaneous insulin absorption: Pilot experiments in pigs

**DOI:** 10.1016/j.curtheres.2026.100846

**Published:** 2026-09-23

**Authors:** Misbah Riaz, Ilze Dirnena-Fusini, Sverre Christian Christiansen, Sven Magnus Carlsen

**Affiliations:** a Department of Clinical and Molecular Medicine, Faculty of Medicine and Health Sciences, Norwegian University of Science and Technology, n - 7491 Trondheim, Norway; b Department of Endocrinology, St. Olav’s Hospital, Trondheim University Hospital, n - 7006 Trondheim, Norway

**Keywords:** Animal studies, Artificial pancreas, Glucagon, Insulin

## Abstract

**Introduction:**

Delayed subcutaneous (SC) insulin absorption remains one of the major barriers to optimal blood glucose control in people with type 1 diabetes and to the development of a fully automated artificial pancreas with SC insulin delivery. Our recent proof of concept study demonstrated that injecting 0.1 mg glucagon at the SC insulin (NovoRapid®) injection site enhances insulin absorption in a porcine model. Present experiments investigate the pharmacokinetics of Lyumjev®, the fastest-acting insulin currently available, when micro-doses of glucagon are co-injected at the same SC site.

**Methods:**

20 anesthetized pigs were administered 10 IU of Lyumjev® SC. Animals were assigned to either a glucagon group (*n* = 12), receiving micro-doses of glucagon at the same subcutaneous injection site as insulin, or a control group (*n* = 8), receiving Lyumjev® only. Arterial blood samples were collected over a 210-minute period to measure glucose, insulin, and glucagon concentrations.

**Results:**

Compared with Lyumjev® alone, the addition of glucagon to Lyumjev® was associated with an approximately 21% higher mean C_max_ (58 vs 48 mU/L) and a 17% earlier T_max_ (25 vs 30 min). Early insulin exposure increased by 109% during the first 15 minutes and by 60% during the first 30 minutes following the addition of glucagon to Lyumjev® compared with Lyumjev® alone (AUC₀–₁₅: 654 vs 313 mU·min/L and AUC₀–₃₀: 1506 vs 942 mU·min/L, respectively).

**Conclusion:**

These experiments in pigs suggest that micro-amounts of glucagon, when injected at the same SC site as insulin, may speed up and enhance the early absorption of a single SC insulin bolus.

## Introduction

People with type 1 diabetes (T1D) depend entirely on exogenous insulin administration due to the auto-immune destruction of the insulin-producing β cells in the pancreas. Despite substantial advances in the treatment modalities, many individuals with T1D struggle to maintain their glucose levels within the desired target ranges, increasing the risk of acute and long-term complications.^1^, ^2^, ^3^

Most individuals with T1D are treated with multiple daily insulin injections (MDII) or continuous SC insulin infusion (CSII) via an insulin pump.^4^ A major obstacle to achieving optimal glucose control is the slow and variable absorption of even the fastest-acting insulin formulations from SC tissue, with an onset of action 10 to 30 minutes after administration, and peak glucose-lowering effects are observed after 1-2 hours.^5^ Due to the delayed glucose-lowering effect, aggressive insulin dosing to limit early postprandial hyperglycemia increases the frequency and severity of late postprandial hypoglycemia.^4^

Many individuals with T1D use a CSII pump and a continuous glucose monitoring (CGM) sensor. This system informs the patients about their blood glucose levels in real-time and enables them to adjust the insulin infusion rate as necessary. However, the present state-of-the-art treatment is an artificial pancreas (AP), i.e., an automated insulin delivery system that uses CGM data to guide insulin administration through a control algorithm.^6^ Currently, only hybrid APs are commercially available, and these systems require meal announcements and carbohydrate counting to calculate insulin boluses. Fully automated APs, which require no patient input, are not yet available.

The reason for exploring a bi-hormonal AP is that it allows more aggressive insulin treatment, as glucagon can be used to treat and prevent hypoglycemia. Although short- and medium-term studies have demonstrated improvements in Time in Range (TIR, glucose 3.9–10.0 mmol/L) with bi-hormonal SC APs, glucose control remains suboptimal.^7^, ^8^, ^9^, ^10^ Suboptimal glucose control, even with a bi-hormonal AP, results from inherent limitations of the dual-SC approach, including delays in SC CGM glucose sensing and, most importantly, the slow absorption and delayed effect of SC-delivered insulin. Insulin absorption in SC tissue is also highly variable and is influenced by factors such as local blood flow, skin temperature, and injection technique.^11^^,^^12^

Our research group (Artificial Pancreas Trondheim, APT) recently demonstrated in healthy subjects that SC micro-doses of glucagon (0.1 mg and 0.01 mg) increased the local SC blood flow up to fivefold.^13^ Based on this, we hypothesized that this principle could be applied to bi-hormonal SC APs by using micro-doses of glucagon in an innovative way to accelerate the absorption of SC insulin meal boluses, while larger glucagon doses could be used to prevent or treat hypoglycemia.^14^ If glucagon facilitates SC insulin absorption, it may also improve the pharmacodynamics of insulin and potentially enable bi-hormonal fully automated AP systems to achieve good glucose control.^15^

Our recent proof-of-concept study in anesthetized pigs demonstrated an increase in the total amount of insulin absorbed when 0.1 mg glucagon was injected simultaneously at the same SC site as insulin aspart (NovoRapid®).^16^ Visually, the insulin concentration–time profiles suggested faster absorption and a 38% higher peak insulin concentration (C_max_), whereas after 2 hours no apparent differences were observed between the groups. The plasma insulin area under the curve (AUC_0-∞_) was significantly larger after glucagon administration. However, no significant differences were observed in time to maximum insulin concentration (T_max_) between the two groups.

We performed a new series of experiments on anesthetized pigs to further investigate our hypothesis. In these proof-of-concept experiments, we used ultra-rapid insulin lispro (Lyumjev®), the fastest-acting insulin formulation available on the market.^17^ This formulation also contains the vasodilating drug Treprostinil (0.00001 mg per unit of insulin) to enhance the absorption, as well as citrate to increase the capillary permeability. We hypothesized that micro-doses of glucagon would further enhance the absorption of insulin lispro in Lyumjev®, despite the presence of formulation-specific absorption-enhancing components in Lyumjev®.

## Materials and methods

### Ethics approval and study design

The animal experiments were approved by the Norwegian Food Safety Authority (FOTS nr. 29687) and were in accordance with the «Norwegian Regulation on the Use of Animals in Research» and the «Directive 2010/63/EU on the protection of animals used for scientific purposes». All experiments were performed following the guidelines of good laboratory practice.

This was a series of open, randomized experiments where interventions and controls were compared. The randomization procedure included the injection side of the neck and the order of intervention.

No formal sample size calculation was performed because this was an exploratory proof-of-concept study. Sample size was based on experience from previous same series of studies and practical considerations regarding animal experimentation.

### Animals and animal handling

We conducted a series of experiments on 20 juvenile, non-diabetic crossbred pigs (50 % Landswine, 50 % Yorkshire) approximately 3 months old. Males (*n* = 17, 35.1 ± 4.9 kg) and females (*n* = 3, 39.0 ± 0.0 kg) were obtained from the same local breeder approximately one week before the trials and acclimatized to the staff and new environment in the animal research facility. Both sexes were included; however, the study was not designed or powered to evaluate sex-specific differences. The pigs were kept in a pen (2.8 m^2^) with a concrete floor covered with wood chips. There was a possible connection to the other bins if more pigs were in the facility (total space 11.2 m^2^). In every bin, a heating lamp was provided. The facility maintained a day-night photoperiod (night: 2100 – 0500, dawn: 0500 – 0700, day: 0700 – 1900, dusk: 1900 – 2100) at 22°C and relative humidity 45 % ± 5 %. The pigs were fed standard food (Format Vekst 100, Felleskjøpet, Norway), and fresh water was available *ad libitum*. Food was withheld for 17 hours before the start of each experiment, and all experiments were initiated in the morning.

The pigs were monitored continuously during the experiments. All surgical procedures, drug administrations (other than premedication/light sedation), and blood samplings were performed under general anesthesia.

### Study procedure

#### Premedication and anaesthesia

The pigs were premedicated with an intramuscular injection of 10 mg/kg azaperone (Separon vet.®, Richter Pharma AG, Austria) and 10 mg/kg ketamine (Ketalar®, Pfizer AS, Norway) and an intravenous (IV) infusion of 1 mg atropine (Takeda AS, Asker, Norway). Anesthesia was induced by an IV infusion of 150-250 µg fentanyl (Actavis Group, Hafnarfjörður, Iceland), 75-125 mg thiopental (VUAB Pharma AS, Roztoky, Czech Republic), and 150-250 mg ketamine before intubation. Anesthesia was maintained by continuous IV infusion of 0.5 mg/kg/hour midazolam (Accord Healthcare Limited, Middlesex, UK) and 7.5 µg/kg/hour fentanyl, with continuous inhalation of 0.5 – 2 % of isoflurane (Baxter AS, Oslo, Norway). An IV infusion of 2 g cephalothin (Villerton Invest SA, Luxembourg) was given as antibiotic prophylaxis immediately after establishing anesthesia and repeated every third hour. The pigs were euthanized while under general anesthesia at the end of the study day with an IV infusion of 100 mg/kg phenobarbital (NAF, Apotek, Lørenskog, Norway).

#### Surgical procedures

Drugs and IV fluids were administered using a venous catheter inserted in the left internal jugular vein. Blood samples were collected through an arterial catheter inserted into the left carotid artery.

A small, low laparotomy was performed to expose the bladder and place a bladder catheter for urine collection.

#### Suppression of endogenous glucagon and insulin secretion

To suppress endogenous glucagon and insulin secretion, the pigs received an initial IV bolus (injected within 10 seconds) of 5 µg/kg octreotide (Sandostatin®, Novartis Europharm Limited, United Kingdom) 30 minutes before the start of glucose infusion, immediately followed by continuous IV infusion of 5 µg/kg/hour octreotide throughout the experiment. Previously, we have verified that this procedure suppresses endogenous glucagon secretion to unmeasurable levels (< 1.5 pmol/L) and, in most cases, porcine insulin to very low levels (< 1.15 mU/L).^16^

#### Euglycemic clamp

At the start of each experiment, the arterial blood glucose levels were stabilized to a normoglycemic target range of 4.5 to 5.5 mmol/L by adjusting the IV infusion rate of a 20 percent glucose solution (Glucos B. Braun, B. Braun Melsungen AG, Germany). According to the protocol, blood glucose concentration had to be stable, defined as ≤ 0.2 mmol/L variance between the last three samples without any glucose infusion rate adjustments the last 15 minutes before starting the intervention.

The glucose infusion rate was adjusted as needed to maintain the glucose levels in the target range of 4.5 to 5.5 mmol/L (euglycemic clamp) throughout the whole 210-minute experimental period.

#### Insulin and micro-glucagon interventions

The experiments were conducted in two series using Lyumjev® insulin, with each series including subgroups based on the amount of glucagon administered with 10 IU of Lyumjev®. In the first series, the control subgroup received only Lyumjev® insulin, while the glucagon subgroups were given 0.1 mg or 0.01 mg doses of glucagon in addition to Lyumjev®. In the second series, the control subgroup received the vehicle solution (contained only additives, and glucagon removal was verified by mass spectroscopy) in addition to Lyumjev®, while the glucagon subgroup received 0.005 mg of glucagon administered twice (at time 0 and 15 minutes) and a dose of 0.00005 mg glucagon was administered in pigs included in series 3. A total of 12 pigs were included in the glucagon groups, and 8 pigs were included in the control groups. The glucagon groups were then subgrouped according to the amount of glucagon. The control group was also further subgrouped into Lyumjev® alone or vehicle (Table 1).

**Table 1:** Overview of glucagon doses and animal characteristics.

Experiment Nr	Insulin used (100 IU/ml)	Groups, (n)	Sex (M/F)	Treatment	Mean weight, kg (SD)
**1**	Lyumjev®	Control (*n* = 5)	3/2	Insulin only	39.4 (3.1)
**1**	Lyumjev®	Glucagon (*n* = 3)	2/1	0.1 mg glucagon	38.0 (0.8)
**1**	Lyumjev®	Glucagon (*n* = 2)	2/0	0.01 mg glucagon	40.2 (3.2)
**2**	Lyumjev®	Glucagon (*n* = 3)	3/0	0.005 mg glucagon × 2*	29.5 (1.9)
**2**	Lyumjev®	Control (*n* = 3)	3/0	vehicle	30.0 (2.4)
**3**	Lyumjev®	Glucagon (*n* = 4)	4/0	0.00005 mg glucagon	35.7 (1.9)

⁎ 0.005 mg glucagon given at time 0 and time 15 minutes.

As the first injection (time point zero), the pigs in the glucagon groups received a 0.1 mL SC glucagon injection (Novo Nordisk A/S, Denmark) (administered over 60 seconds), reconstituted in 0.9 % NaCl solution to the required concentration. One minute after the start of the SC glucagon injection, a bolus of 10 IU (0.1 mL) insulin lispro 100 IU/ml (Lyumjev®, Eli Lilly & Co., USA) was injected (time point one minute) during 60 seconds at the exact same SC site. Varying doses of glucagon were used across different groups, as delineated in Table 1, ranging from 0.1 to 0.00005 mg. Regardless of the dosage, the glucagon solutions' volume remained constant at 0.1 mL. The pigs in the control group received only 10 IU (0.1 mL) of Lyumjev® in 60 seconds from time point zero, i.e., the insulin injection concluded one minute earlier in pigs in the control groups than the pigs in the glucagon groups.

The glucagon and insulin infusions were done via separate tubes using a Chemyx Fusion 100 syringe pump. To ensure that the infusions were given at the exact same SC site, we used a custom-made insertion guide with a needle depth of 9 mm (Figs. 1a and b). The injection guide precision was tested on a silicon phantom and dead pigs to verify that the injections were done exactly at the same SC site (Fig. 1b). Using an injection guide was of utmost importance, as we suspected from experiments on us that glucagon's SC vasodilatory effect is highly local. Injections for all study pigs were performed in SC adipose tissue on the neck behind the ear, while the side of the neck was randomized.

**Fig. 1:**
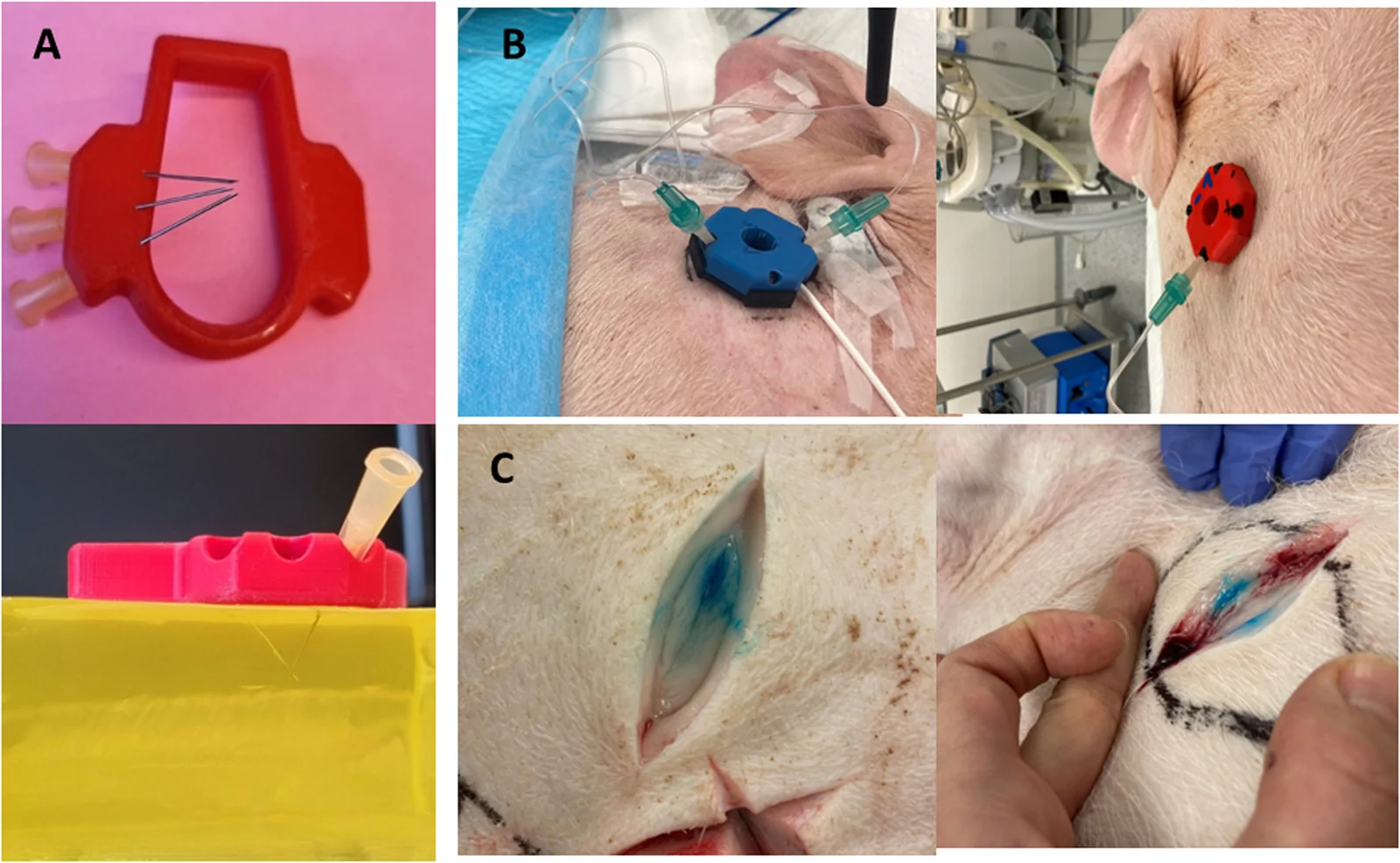
(A) Custom-made injection guide used to ensure precise co-localized subcutaneous administration of insulin and glucagon. (B) Experimental insulin infusion and blood sampling setup. (C–D) Verification of injection accuracy in postmortem pigs demonstrating consistent co-localized delivery of insulin and glucagon to the same subcutaneous site.

#### Blood sampling

The first blood sample for glucose measurement was drawn seconds before the octreotide bolus was administered. Afterward, samples for glucagon, insulin and glucose analyses were collected at the time points 0, 1, 5, 10, 15, 20, 25, 30, 35, 40, 50, 60, 90, 120, 150, 180, and 210 minutes. Further, additional blood glucose samples to adjust euglycemic clamp infusion were drawn when necessary. To cover at least two predicted half-lives of insulin lispro, the length of the total experiment was set to last for 210 minutes.

#### Sample handling and analysis

Arterial blood samples were immediately analysed for glucose. Analyses were performed in the heparinized syringes using a Radiometer ABL 800 FLEX blood gas analyser.

Arterial blood samples for hormone analyses were collected into 3 mL EDTA vacutainers and kept on ice for 10 minutes before centrifugation. Following that, plasma was transferred to two Eppendorf tubes and stored at −18°C for the duration of the experiment day, and thereafter, transferred to a −80°C freezer until analysis, that was performed within the following week.

Insulin concentrations were determined using Iso-Insulin ELISA kit 10-1128-01 (Mercodia, Uppsala, Sweden), with a lower detection limit of 2.0 mU/L, according to the manufacturer. In the results, all values below the detection limit (< 2.0 mU/L) are set as 1.00 mU/L. Glucagon concentrations were determined using Glucagon ELISA kit 10-1271-01 (Mercodia, Uppsala, Sweden), with a lower detection limit of 1.5 pmol/L as determined according to FDA/EMA guidelines. In the results, all values < 1.5 pmol/L are set as 0.75 pmol/L. The plasma glucagon concentrations were only investigated in the Lyumjev®/0.1 mg glucagon and Lyumjev®/0.01 mg glucagon groups, where the highest doses of glucagon were used.

All insulin and glucagon samples were run in doubles, and the mean value of the two measurements was used. The ELISA kits for iso-insulin and glucagon were all from the same batch. The analyses were carried out by the same engineer (IDF) in the same setting. Intra-assay and inter-assay variation for both iso-insulin and glucagon were < 7 %. One pig (male) (0.00005 mg glucagon subgroup) was excluded from further analyses. Each animal was considered an independent biological replicate (glucagon group, *n* = 11; control group, *n* = 8). Blood samples collected at different time points represented repeated measurements within the same animal. Each plasma sample was analyzed in duplicates, representing technical replicates. The mean of the duplicate measurements was used as the plasma concentration value for each animal at each time point and for subsequent statistical analyses. Reported n-values therefore refer to the number of independent animals (Table 1).

### Data analysis

#### Pharmacokinetic analysis

C_max_ (mean maximum insulin concentration) and T_max_ (mean time to peak insulin concentration) were obtained directly from the measured insulin concentrations in plasma. Individual Areas Under the Curve (AUC) were calculated from measured mean insulin concentrations in blood plasma using the linear trapezoidal method in GraphPad Prism 10. AUC_0-210_ is a measure of total systemic exposure to insulin until 210 minutes post-injection.

#### Statistical analysis

All insulin levels and glucose infusion rates were adjusted for baseline values and standardized to a body weight of 40 kg. The mean and range of plasma insulin concentrations, glucose infusion rates, AUC_0-15_ to AUC_0-210_ of insulin, and glucose infusion rates were calculated using GraphPad Prism 10. Potential statistical differences in insulin concentration over time, C_max_, T_max_, and AUCs between the glucagon and control groups were tested using a Mann-Whitney U test. Nonparametric methods were selected because of the limited sample sizes and uncertainty regarding normality assumptions. Due to the exploratory nature of the study and the small subgroup sample sizes, no formal statistical comparisons were performed between subgroups. Data are presented as means with ranges or means with 95% confidence intervals. Data was plotted using GraphPad Prism version 10.

## Results

### Exclusions

One pig (male) assigned to the 0.00005 mg glucagon group was excluded from all the analyses due to documented failure in insulin infusion delivery. This was confirmed by assessing the glucose infusion rate and the absence of detectable insulin in subsequent plasma samples.

### Overview of groups and animal characteristics

The mean body weight and number of pigs included in each experimental sub-group are presented in Table 1.

### Pharmacokinetic parameters of insulin (pooled data)

Pharmacokinetics of insulin (mean C_max_, mean T_max_, and mean AUCs) for the control and glucagon groups are summarised in Table 2. Mean insulin concentrations over time and corresponding AUCs for each group, adjusted for baseline insulin level and adjusted to a weight of 40 kg, are presented in Fig. 2.

**Table 2:** Pharmacokinetic parameters of insulin (pooled data).

Groups	T_max_ (mins)	Mean (SD) C_max_ (mU/L)	AUC_0-15_ (mU*min/L) (95%CI)	AUC_0-30_ (mU*min/L) (95%CI)	AUC_0-210_ (mU*min/L) (95%CI)
**Glucagon** **(*n*=11)**	25	58 (23)	654 (434 to 874)	1506 (1158 to 1854)	4339 (3801 to 4876)
**Control** **(*n*=8)**	30	48 (13)	313 (265 to 362)	942 (783 to 1101)	3604 (3210 to 3999)
***P*-Values**	0.0004	0.18	0.0008	0.02	0.051
**Absolute Difference** **(95% CI)**	−5	10	341 (119 to 563)	564 (198 to 930)	735 (115 to 1355)
**Relative Difference (%)** **(95%CI)**	−17	21	109 (38 to 180)	60 (21 to 99)	20 (3 to 38)

Absolute differences are calculated as mean glucagon minus mean control. Relative differences were calculated as (glucagon − control)/control × 100. Confidence intervals for relative differences were derived from the corresponding confidence intervals for the absolute differences.

**Fig. 2:**
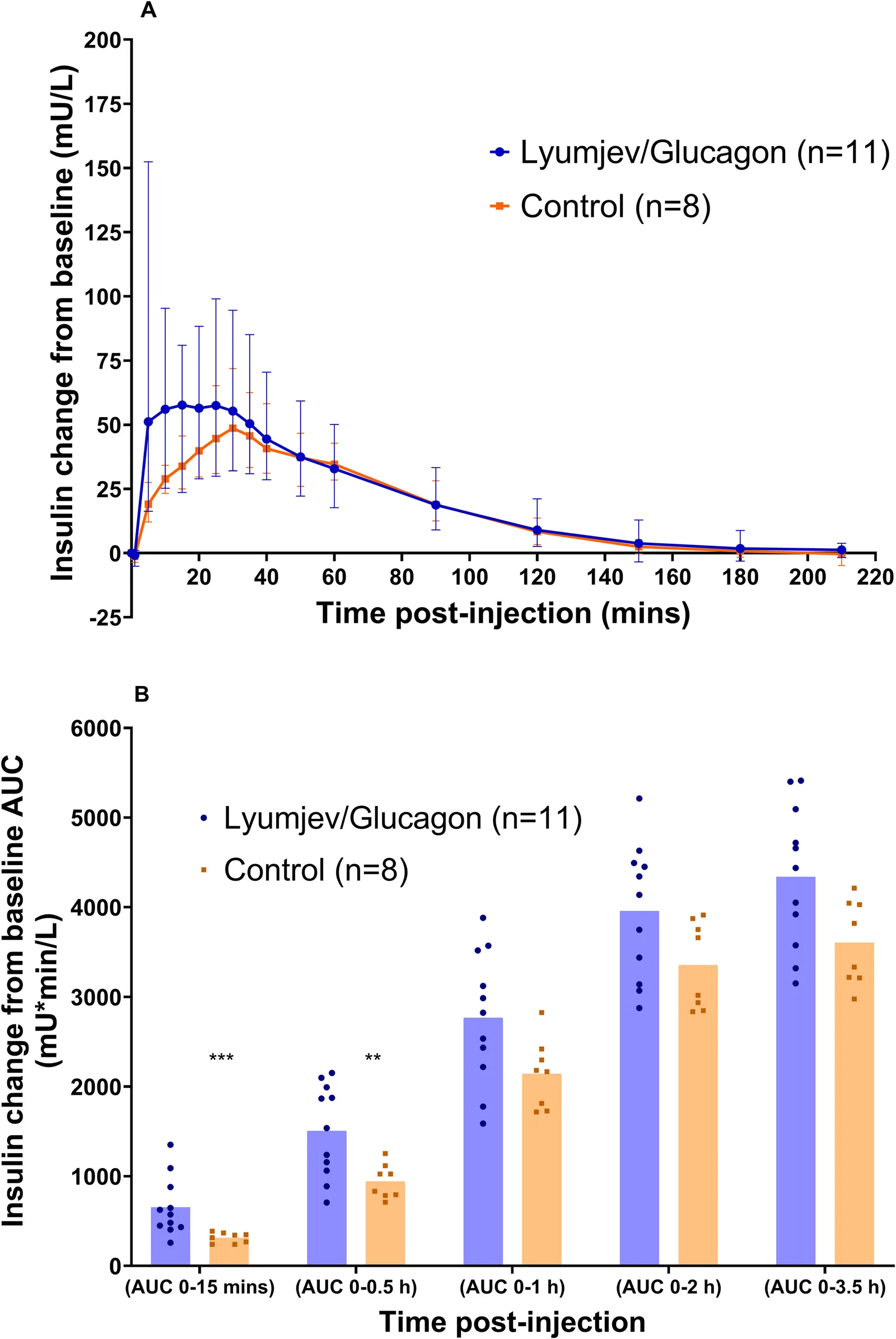
Mean (range) insulin concentrations change from baseline in arterial plasma over time in the glucagon group and the control group (A). Insulin AUC, the area under the insulin concentration versus time curve for the glucagon group and the control group (B). Dots in the AUC graph represent individual pigs. Individual pigs represented biological replicates (n); plasma samples were measured in duplicate as technical replicates. * = P<0.05, ** =P<0.001, *** = P<0.0001.

Compared with the control group (Lyumjev® plus vehicle), the glucagon group (Lyumjev® plus glucagon) demonstrated a 17% earlier mean insulin T_max_ (25 vs 30 minutes) and a 21% higher mean C_max_ (58 vs 48 mU/L) (Table 2).

Early insulin exposure increased by 109% during the first 15 minutes (AUC₀-₁₅: 654 vs 313 mU·min/L) and by 60% during the first 30 minutes (AUC₀-₃₀: 1506 vs 942 mU·min/L) following co-localized glucagon administration as compared to the control group (Table 2). This was reflected by higher mean insulin concentrations during the early absorption phase, particularly at 5, 10 and 15 minutes after injection (Fig. 2).

### Pharmacokinetic parameters of insulin for each subgroup

Pharmacokinetics of insulin (mean C_max_, mean T_max_, and mean AUCs) for each subgroup, adjusted for baseline values and standardized to a weight of 40 kg, are summarised in Table 3. Mean insulin concentrations over time for each subgroup, adjusted for baseline values and adjusted to a weight of 40 kg, are presented in Figs. 3a and 3b, whereas AUCs for each subgroup are presented in Figs. 3c and 3d. Due to the limited sample sizes in the subgroups, these data should be considered descriptive and exploratory rather than confirmatory.

**Table 3:** Pharmacokinetic parameters of insulin in all subgroups.

Groups	T_max_ (mins)	Mean (SD) C_max_ (mU/L)	AUC_0-15_ (mU*min/L) (95%CI)	AUC_0-30_ (mU*min/L) (95%CI)	AUC_0-210_ (mU*min/L) (95%CI)
**Control (*n*=5)**	35	44 (7)	315 (259 to 371)	893 (781 to 1005)	3476 (3076 to 3875)
**Vehicle (*n*=3)**	30	60 (11)	311 (239 to 383)	1026 (863 to 1184)	3761 (3355 to 4166)
**Glucagon 0.1 mg (*n*=3)**	25	71 (26)	560 (450 to 670)	1546 (1288 to 1805)	4512 (3971 to 5052)
**Glucagon 0.01 mg (*n*=2)**	25	66 (34)	548 (405 to 690)	1468 (1089 to 1848)	4357 (3623 to 5090)
Glucagon 0.005 mg × 2*(*n*=3)	10	63 (34)	742 (359 to 1125)	1598 (1096 to 2100)	3794 (3103 to 4485)
**Glucagon 0.00005 mg (*n*=3)**	5	69 (72)	730 (213 to 1246)	1398 (857 to 1940)	4683 (3925 to 5441)

⁎ 0.005 mg glucagon given at time 0 and time 15 minutes.

**Fig. 3:**
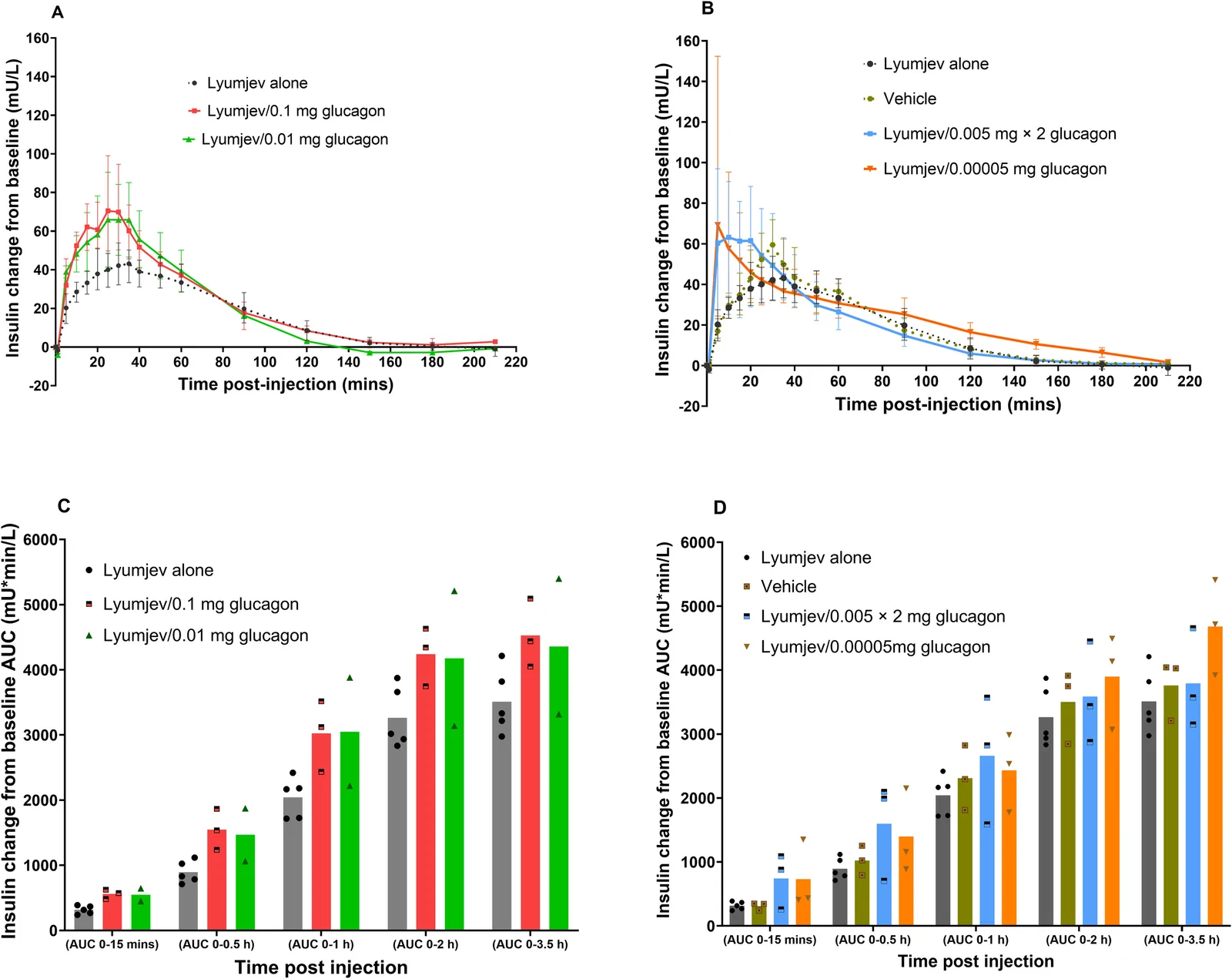
Mean (range) insulin concentrations in arterial plasma over time in all experimental subgroups (a and b). Insulin AUC (CI), area under the concentration versus time curve for all experimental subgroups (c and d). Dots in the AUC graphs represent individual pigs. Individual pigs represented biological replicates; plasma samples were measured in duplicate as technical replicates.

### Euglycemic clamp and glucose infusion (pooled data)

Mean glucose infusion rates and AUC_0-15_ to AUC_0-210_ are shown in Fig. 4. Mean maximum glucose infusion rates, time of maximum glucose infusion rates, and AUCs are summarised in Table 4. Estimated differences in glucose infusion rates and corresponding AUCs between pooled glucagon and control groups were modest (Table 4).

**Fig. 4:**
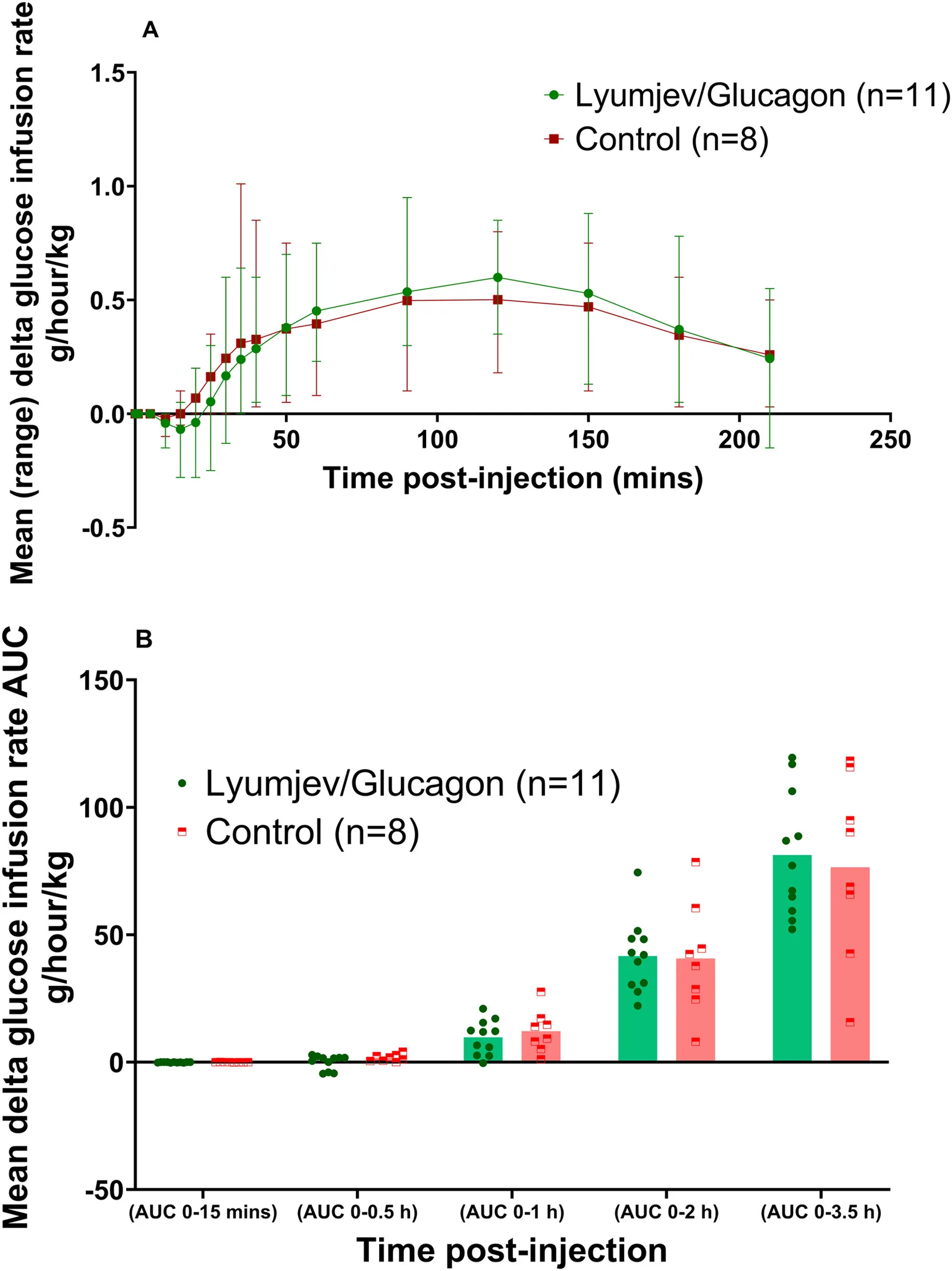
Mean (range) glucose infusion rate over time in the glucagon group and the control group (A). Glucose infusion AUC, the area under the glucose infusion rate versus the time curve for the glucagon group and the control group (B). Dots in the AUC graph represent individual pigs. Individual pigs represented biological replicates (n); plasma samples were measured in duplicate as technical replicates.

**Table 4:** Pharmacodynamic parameters (pooled data).

Groups	Time (mins)	Mean (SD) Max Glucose Infusion (g/hr/kg)	AUC_0-15_ (g/hr/kg) (95%CI)	AUC_0-30_ (g/hr/kg) (95%CI)	AUC_0-210_ (g/hr/kg) (95%CI)
**Glucagon (*n*=11)**	120	0.59 (0.16)	0.38 (−1.08 to 0.32)	−0.06 (−2.1 to1.9)	81.85 (62.33 to 101.40)
**Control (*n*=8)**	120	0.50 (0.20)	0.09 (−0.35 to 0.16)	1.67 (0.23 to 3.10)	76.20 (54.50 to 97.89)
***P*-values**	0.81	0.34	0.28	0.26	>0.99

Mean glucose infusion rates in all subgroups, adjusted for baseline values and standardized to a weight of 40 kg, are presented in Figs. 5a and 5b, whereas AUCs for each subgroup are presented in Figs. 5c and 5d. Mean maximum glucose infusion rates, time of maximum glucose infusion rates, and AUCs (area under the glucose infusion rate time curve) for subgroups are summarised in Table 5.

**Fig. 5:**
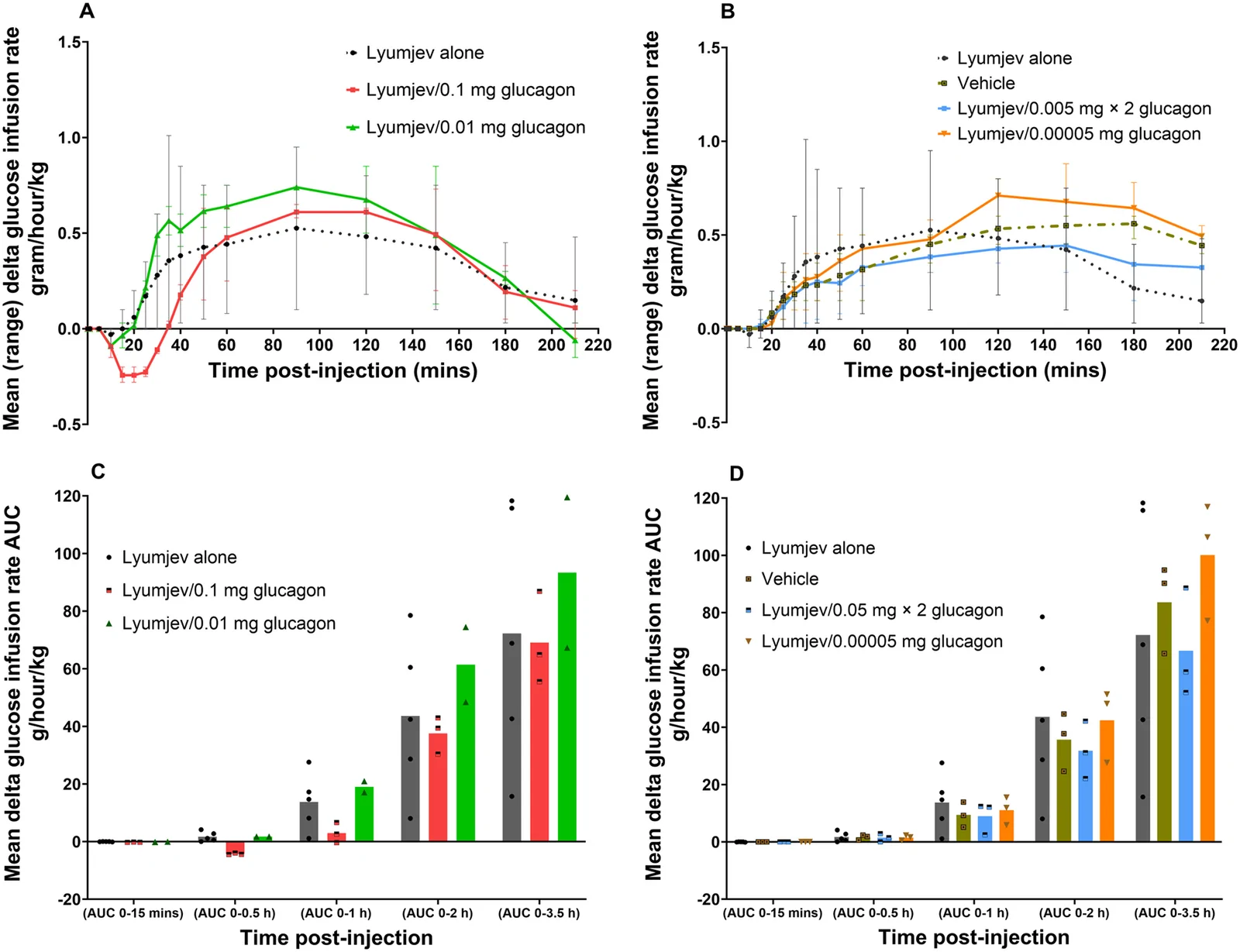
Mean (range) glucose infusion rate over time in all experimental subgroups (a and b). AUC (CI), area under the glucose infusion rate versus time curve for all experimental subgroups (c and d). Dots in the AUC graphs represent individual pigs. Individual pigs represented biological replicates; plasma samples were measured in duplicate as technical replicates.

**Table 5:** Mean glucose infusion rates, time of maximum glucose infusion rate, and AUCs (area under the glucose infusion versus time curve).

Groups	Time (mins)	Mean (SD) Max Glucose Infusion (g/hr/kg)	AUC_0-15_ (g/hr/kg) (95%CI)	AUC_0-30_ (g/hr/kg) (95%CI)	AUC_0-210_ (g/hr/kg) (95%CI)
**Lyumjev**® **control (*n*=5)**	90	0.53 (0.34)	−0.15 (0.00 to −0.45)	1.70 (0.00 to 3.44)	71.75 (46.84 to 96.65)
**Vehicle (*n*=3)**	180	0.56 (0.07)	0.00 (0.00 to 0.00)	1.63 (0.71 to 2.54)	83.62 (74.56 to 92.67)
**0.1 mg glucagon (*n*=3)**	90	0.61 (0.04)	−1.08 (−1.54 to −0.61)	−4.31 (−4.92 to −3.70)	68.96 (53.82 to 84.10)
**0.01 mg glucagon (*n*=2)**	90	0.74 (0.29)	−0.54 (−0.99 to −0.07)	1.75 (0.74 to 2.76)	92.98 (65.78 to 120.20)
**0.005 mg × 2 glucagon (*n*=3)**	150	0.44 (0.15)	0.04 (0.00 to 0.18)	1.46 (0.11 to 2.81)	66.69 (53.60 to 79.79)
**0.00005 mg glucagon (*n*=3)**	120	0.71 (0.14)	−0.18 (−0.46 to 0.10)	1.46 (0.39 to 2.52)	100.10 (86.79 to 113.40)

*0.005 mg glucagon given at time 0 and time 15 minutes.

### Pharmacokinetic parameters of glucagon

The plasma glucagon concentration was below the lower limit of detection (1.5 pmol/L) at time zero in all pigs except two, one in the 0.1 mg glucagon group (17.4 pmol/L) and one in the 0.01 mg glucagon group (13.2 pmol/L).

In the Lyumjev® + 0.1 mg glucagon and Lyumjev® + 0.01 mg glucagon subgroup, the mean T_max_ of glucagon was 15 minutes and 10 minutes, respectively, whereas mean C_max_ was 150.4 pmol/L and 11.4 pmol/L, accordingly. No glucagon data was collected for the 0.005 × 2 mg and 0.00005 mg glucagon groups. Plasma glucagon concentration was below the lower limit of detection (1.5pmol/L) at all time points in all pigs in the control group (Fig. 6).

**Fig. 6:**
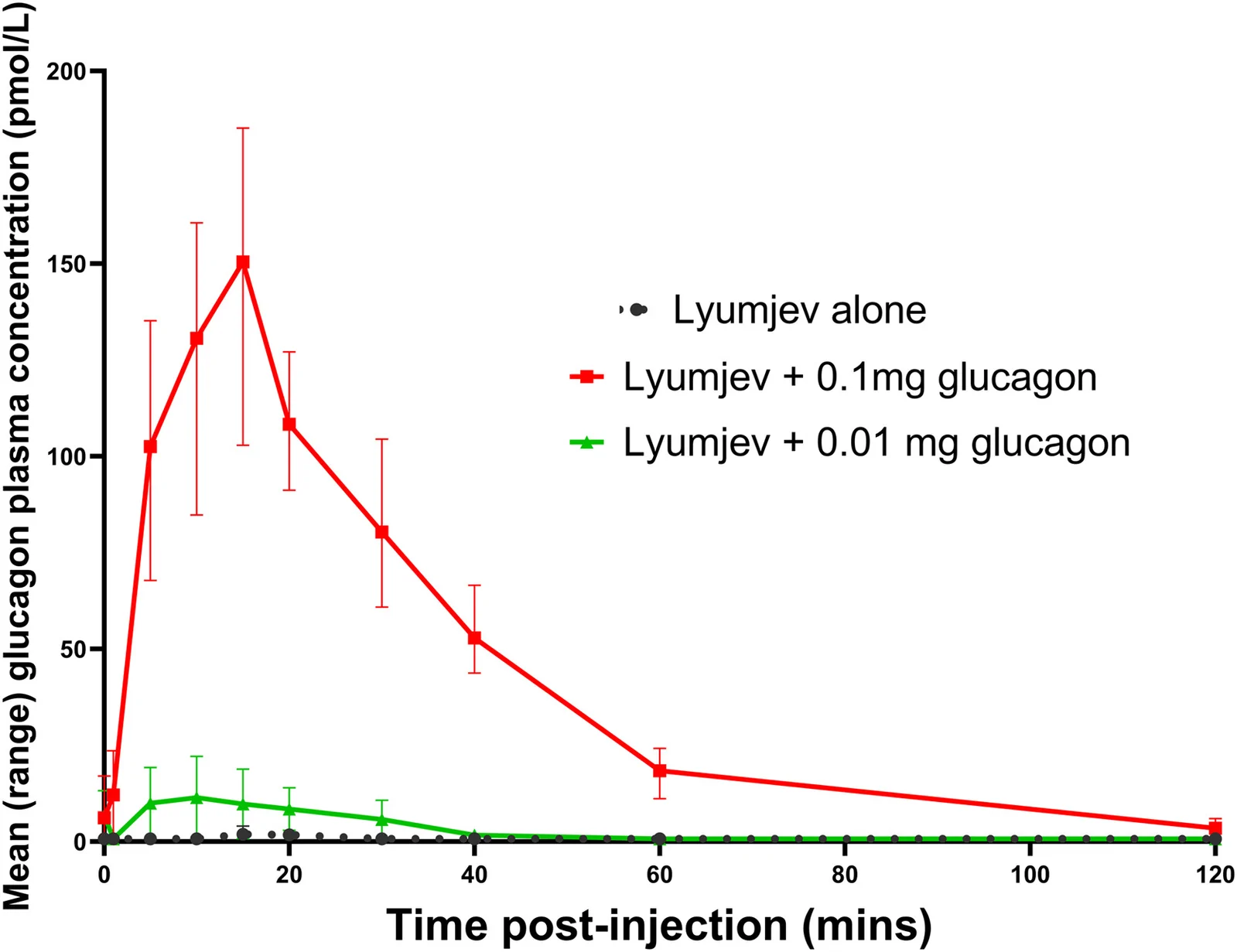
Mean (range) plasma glucagon concentrations over time following subcutaneous administration of 0.1 mg glucagon, 0.01 mg glucagon, and control treatment subgroups. Individual pigs represented biological replicates; plasma samples were measured in duplicate as technical replicates.

## Discussion

The principal finding of this proof-of-concept study is that simultaneous administration of glucagon at the site of insulin injection may enhance early SC insulin absorption. The most notable observations were 17% shorter mean T_max_, a 21% higher mean C_max_, a 109% increase in AUC_0-15_, and a 60% increase in AUC_0-30_. Total insulin exposure over the entire study period was also higher (20% increase in AUC_0-210_), although the largest estimated effects were observed during the early absorption phase. From a clinical point of view, increased insulin absorption during the first 15 minutes and 30 minutes is the most interesting observation. A major challenge for people with T1D is achieving a sufficiently rapid onset of insulin action following meal-time insulin boluses. Thus, these improvements in early pharmacokinetic profile of insulin may also translate into a more rapid onset of insulin action and improved postprandial glucose control. Whether these pharmacokinetic changes translate into clinically meaningful pharmacodynamic benefits remains to be established in future human studies. Our current experiments were primarily designed to study insulin pharmacokinetics. Euglycemic clamp investigations were added mainly to look for potential metabolic effects of glucagon administration and to monitor possible adverse effects as observed in our previous study with larger glucagon doses.^14^ In the present study, glucagon doses ranging from as high as 0.1 mg to as low as 0.00005 mg per 10 IU of insulin were used. The larger glucagon doses (0.1 mg and 0.01 mg) were associated with an initial reduction in glucose infusion requirements, consistent with transient glucagon-mediated hepatic glucose secretion. At lower glucagon doses, estimated differences in glucose infusion requirements were smaller; however, interpretation is limited by the exploratory study design and limited subgroup sample sizes (Table 5 and Fig. 5). No differences in mean glucose infusion rates were observed between the pooled glucagon and control groups. Furthermore, more than half of the animals in the glucagon group received significantly smaller glucagon doses, suggesting that smaller doses of glucagon may not adversely affect glucose metabolism. On the other hand, for 0.1 mg glucagon, the largest glucagon dose, the glucose infusion had to be completely shut off during the first 5 to 35 minutes following injection, indicating a clear adverse effect of higher glucagon doses.

It is well established that the absorption of SC-injected insulin depends, among other factors, on the local SC blood flow. Local SC blood flow increases with exercise, local massage, a warmer environment, local skin heating and other physiological factors at the injection site.^12^^,^^18^ We previously reported that local abdominal SC blood flow in healthy subjects increased several hundred percent following SC injection of 0.1 mg or 0.01 mg glucagon, as measured by laser doppler flowmetry (LDF).^13^ Although local SC blood flow was not measured in the present study, the observed acceleration of insulin absorption is consistent with the hypothesis that glucagon enhances SC insulin absorption through local vasodilation.^14^^,^^15^

However, one-third of the subjects in our previous human trial^13^ were non-responders, possibly attributable to LDF's limited blood flow measuring area and potential misalignment of glucagon injections and LDF measurement area. To improve the accuracy of our injection site, we recently modified the LDF probe by drilling an injection hole 1 mm from the point where the probe emits light and measures blood flow. Exploratory experiments performed with modified probe (conducted to gather data on the modified probe and devise protocols for future studies) on our group members (not to be formally published) demonstrated that 0.0005 mg and 0.00005 mg doses of glucagon increased the median blood flow up to a 1000% compared with the injection of the same volume of a 0.9 % saline solution. However, these observations have not been formally investigated and should therefore be interpreted with caution. Considerable interindividual variability in the vasodilatory response was observed, which may explain the wide variation in insulin responses observed in the present pig experiments (Fig. 2, Fig. 3, Fig. 4), where individual animal data are presented. Despite this variability, local vasodilatory responses were consistently observed with the modified setup unlike in our previous human trial^13^, suggesting that precise co-localized delivery is crucial for achieving a glucagon effect on insulin absorption. In this context, it should be noted that the present pig experiments used an improved custom-made introducer to secure injection of insulin and glucagon on the exact same SC site.

Lyumjev® was used in present experiments, and it contains 0.00001 mg of the vasodilator Treprostinil per unit of insulin to promote local vasodilation. As stated previously, the present findings suggest that glucagon enhances insulin absorption by a local vasodilatory response. It is therefore noteworthy that increased insulin absorption and early insulin exposure were observed with glucagon despite the presence of a vasodilatory excipient in Lyumjev®. These observations suggest that glucagon may exert an additional local vasodilatory effect despite the presence of treprostinil in Lyumjev®.

People with diabetes using different formulations of fast-acting insulins often complain of local burning or SC injection site irritation, which negatively affects treatment adherence. No adverse effects of local SC glucagon injections were reported in our 22 healthy humans' study where we investigated the vasodilative effects of glucagon on local SC blood flow.^13^

As mentioned above, considerable inter-animal variation in insulin absorption was observed following glucagon administration in pigs. Similar interindividual variation in the vasodilative response to glucagon was also reported in our human trial.^13^ The mechanisms leading to variation in glucagon vasodilatory response remain unknown. Although higher insulin responses were observed in several male pigs, the study was not designed or powered to evaluate sex-related differences, and this observation should therefore be interpreted cautiously. However, this possible sex difference led us to include only male pigs towards the end of the series of experiments. Estrogen is reported to induce changes in glucagon secretion, insulin secretion, and insulin sensitivity.^12^^,^^19^, ^20^, ^21^ Whether estrogen also modifies glucagon's vasodilative effects and consequently its ability to enhance SC insulin absorption in different hormonal states such as pregnancy, menstruation, and menopause, needs to be investigated in future human trials. Notably, sex was not reported as a factor contributing to variation in glucagon’s vasodilative response in our previous human trial.^13^

A valid concern regarding the use of glucagon to enhance insulin absorption is its potential to raise blood glucose concentrations and counteract insulin effects on glucose metabolism as observed with our larger glucagon doses of 0.1 and 0.01 mg. The present findings suggest that the smallest glucagon doses may have no effect on glucose homeostasis. However, the available data are insufficient to exclude modest metabolic effects. Confirmation in adequately powered human studies is therefore required. It should be noted that glucagon is present in human circulation, and that plasma levels increase following a meal because of glucagon release from the intestines both in healthy subjects and in patients with T1D. This effect is absent when equal amounts of glucose are administered intravenously.^22^, ^23^, ^24^, ^25^ In the present pig experiments, endogenous glucagon was effectively suppressed by continuous somatostatin analogue infusion and parenteral glucose infusion, which was confirmed by plasma glucagon measurements. Glucagon increases plasma glucose concentrations primarily by increasing hepatic glucose output. As previously reported by our research group, glucagon undergoes a considerable first-pass metabolism of glucagon in the liver, and this first-pass effect may be saturable.^26^^,^^27^ This suggests that combining micro-doses of glucagon with mealtime insulin injections will have minimal adverse effect on postprandial glucose metabolism in humans, as the liver will already be saturated with glucagon released from the intestine and delivered to the liver at a high concentration via the portal vein. Additionally, circulating glucagon concentration was highest in the 0.1 mg glucagon group, which decreased with a lower dose of 0.01 mg (Fig. 4) and would likely be undetectable with doses of 0.005 mg or 0.00005 mg.

Based on the present data, glucagon enhanced SC insulin absorption is a fascinating possibility and provides the rationale for a proof-of-concept study in humans. Future studies should also investigate whether micro-doses of glucagon similarly enhance the absorption of lispro formulations other than Lyumjev®, other insulin analogues formulations, and human insulin formulations. Based on our experience and currently available human data, we do not anticipate any acute local or systemic adverse effects of using micro-amounts of glucagon to enhance insulin absorption.

## Limitations

The use of anaesthetized pigs in this study is a major limitation as the use of anesthetics may alter physiological processes such as metabolism and circulation^28^, and consequently it might also affect insulin's pharmacokinetic and pharmacodynamic profile. The experiments on awake pigs would probably have provided more reliable data. However, we deemed it unethical not to anaesthetize the pigs because the experiments required frequent blood samplings, precise SC drug administrations, and repeated interventions, procedures that would otherwise expose the animals to unacceptable and unnecessary stress, fear, and pain. The small sample size, with as few as two pigs in one subgroup, is another major limitation. The sample size was kept small due to animal welfare considerations and the cost of the experiments. Furthermore, no prior data on the effect of glucagon on SC Lyumjev® absorption was available, so we could not perform any power calculations. However, it should be noted that all observations favored early enhanced insulin absorption by using glucagon at the site of SC insulin injections. Further, we did not perform any statistics on the subgroups due to the small sample size.

A methodological strength of the present study is the use of a custom-made injection guide that ensured precise co-localized SC delivery of insulin and glucagon. Because the proposed vasodilatory effect of glucagon is expected to be highly localized, accurate placement of both insulin and glucagon at the same SC site was considered essential for maximizing their interaction and minimizing variability associated with injection technique. Another major strength of the present experiments is that endogenous glucagon secretion was totally suppressed in almost all pigs, facilitating the observation of a possible adverse effect of glucagon micro-doses glucose metabolism under control conditions. Endogenous insulin secretion was also suppressed to a very low level by continuous octreotide infusion, allowing assessment of exogenously administered insulin. The absence of obvious differences in glucose infusion rates between subgroups receiving the smallest glucagon doses and the control subgroups suggests that, under the present experimental setup, we cannot not rule out an adverse effect of even the smallest glucagon doses on glucose metabolism. These findings support the hypothesis that glucagon micro-doses may represent a novel strategy for accelerating SC insulin absorption and thereby may contribute to future development of fully automated bi-hormonal AP systems with improved postprandial glucose control.

## Conclusions

The results of this series of proof-of-concept experiments suggest that micro-amounts of glucagon injected at the same SC site as insulin increase the absorption of SC injected insulin in a pig model, even when combined with the ultra-rapid insulin formulation Lyumjev®. However, the effect on glucose homeostasis is unsettled, due to limitations of the experimental set up. Given the persistent challenge of delayed insulin absorption even with ultra-rapid insulin formulations, the potential use of microdoses of glucagon to increase insulin absorption should be studied in humans.

## Author contributions

All authors made substantial contributions to the work prior to submission and have read and approved the final manuscript. MR and IDF planned and performed the experiments. MR analyzed and interpreted the data and wrote the initial draft of the manuscript. IDF contributed to writing the Methods section and reviewed the manuscript. SMC conceived the idea for the study, supervised the experiments, was involved in data interpretation, and reviewed the manuscript. SCC was involved in supervision of the experiments, data interpretation, and review of the manuscript.

## Funding statement

The study was funded by the Research Council of Norway (grant number 332850) and the Central Norway Regional Health Authority (grant number 2020/39645).

## Ethics declaration

This study was conducted in accordance with the ARRIVE (Animal Research: Reporting of In Vivo Experiments) guidelines. This study was approved by the Norwegian Food Safety Authority.

## Data availability statement

The data supporting this study's findings are available from the corresponding author upon reasonable request.

## Declaration of competing interest

The authors declare that the research at the time was conducted without any commercial or financial relationships that could be construed as a potential conflict of interest. NTNU, the university where the research was conducted and where the researchers reside, has a patent filed related to the research. The authors Sven Magnus Carlsen and Sverre Christian Christiansen are among the inventors. The remaining authors have no conflicts of interest to declare.
